# Discovery of Two Novel Viruses of the Willow-Carrot Aphid, *Cavariella aegopodii*

**DOI:** 10.3390/v16060919

**Published:** 2024-06-05

**Authors:** Gaoyang Jiao, Zhuangxin Ye, Kehui Feng, Chuanxi Zhang, Jianping Chen, Junmin Li, Yujuan He

**Affiliations:** State Key Laboratory for Managing Biotic and Chemical Threats to the Quality and Safety of Agro-Products, Key Laboratory of Biotechnology in Plant Protection of MARA and Zhejiang Province, Institute of Plant Virology, Ningbo University, Ningbo 315211, Chinajianpingchen@nbu.edu.cn (J.C.); lijunmin@nbu.edu.cn (J.L.)

**Keywords:** *Cavariella aegopodii*, aphid, RNA virome, high-throughput sequencing, RNA interference pathway

## Abstract

The advancement of bioinformatics and sequencing technology has resulted in the identification of an increasing number of new RNA viruses. This study systematically identified the RNA virome of the willow-carrot aphid, *Cavariella aegopodii* (Hemiptera: Aphididae), using metagenomic sequencing and rapid amplification of cDNA ends (RACE) approaches. *C. aegopodii* is a sap-sucking insect widely distributed in Europe, Asia, North America, and Australia. The deleterious effects of *C. aegopodii* on crop growth primarily stem from its feeding activities and its role as a vector for transmitting plant viruses. The virome includes Cavariella aegopodii virga-like virus 1 (CAVLV1) and Cavariella aegopodii iflavirus 1 (CAIV1). Furthermore, the complete genome sequence of CAVLV1 was obtained. Phylogenetically, CAVLV1 is associated with an unclassified branch of the *Virgaviridae* family and is susceptible to host antiviral RNA interference (RNAi), resulting in the accumulation of a significant number of 22nt virus-derived small interfering RNAs (vsiRNAs). CAIV1, on the other hand, belongs to the *Iflaviridae* family, with vsiRNAs ranging from 18 to 22 nt. Our findings present a comprehensive analysis of the RNA virome of *C. aegopodii* for the first time, offering insights that could potentially aid in the future control of the willow-carrot aphid.

## 1. Introduction

With the advent of advanced sequencing technologies, particularly metagenomic sequencing, there has been a surge in the discovery of novel RNA viruses in various insect species [1,2]. Unlike vector-borne or plant viruses, these insect specific viruses (ISVs) are incapable of infecting vertebrates or plants, and have been classified into families such as *Togaviridae*, *Reoviridae*, *Rhabdoviridae*, *Dicistroviridae*, *Flaviviridae*, *Iflaviridae*, *Aliusviridae*, *Chuviridae*, and *Negevirus* [1,3,4,5].

*Cavariella aegopodii* (Hemiptera: Aphididae), also known as willow-carrot aphid, is a sap-sucking insects widely distributed in Europe, Asia, North America, and Australia [6,7,8]. This polyphagous species infests a variety of plants, including willow trees, carrot plants, and various other herbaceous plants [6]. The deleterious effects of *C. aegopodii* on crop growth primarily stem from its feeding activities and its role as a vector for transmitting plant viruses, such as carrot red leaf virus (CRLV) and carrot mottle virus (CMotV). These two viruses are persistent and circulative in *C. aegopodii*. After *C. aegopodii* feeding the viruses, the viruses are inhaled into the intestine with saliva, penetrate through the intestinal wall into the hemolymph, and finally return to the salivary glands. And the acquisition access time was at least 30 min for *C. aegopodii*. In addition, they cannot be transmitted vertically to offspring [9]. 

Chemical insecticides are currently crucial for managing population dynamics. However, there is currently no high-quality reference genome available for *C. aegopodii*, and only a few viruses infecting *C. aegopodii* have been reported.

*Iflaviridae* is a family of compact, non-enveloped viruses harboring single-stranded, positive-sense, non-segmented RNA genomes. These genomes span approximately 9–11 kb and contain a sole extensive open reading frame (ORF) which is responsible for encoding a polyprotein of around 3000 amino acids [10]. The family *Iflaviridae* belongs to the order Picornavirales and currently includes only one genus, *Iflavirus*, which comprises 16 species, based on the data from International Committee on Taxonomy of Viruses (ICTV) (https://ictv.global/report/chapter/iflaviridae/iflaviridae, accessed on 5 April 2024) [11]. All Iflavirus species have been isolated from arthropods, primarily insects. These infections may be asymptomatic or may lead to developmental abnormalities, changes in behavior, and the premature death of the host.

*Virgaviridae* is a family of plant viruses characterized by non-enveloped, rod-shaped virions. They possess a single-stranded, positive-sense RNA genome ranging from approximately 6.3 to 13 kb, featuring a 3′-tRNA-like structure and lacking a polyA tail. *Virgaviridae* is subdivided into seven genera as follows: *Garavirus*, *Furovirus*, *Hordeivirus*, *Pecluvirus*, *Pomovirus*, *Tobamovirus*, and *Tobravirus* [12,13]. Most of these viruses encode movement proteins (MPs), which can be either ‘30K’ single proteins or ‘triple gene block’ types, depending on the genus. *Virgaviridae* are predominantly found in a wide range of herbaceous and monocotyledonous plants, as well as in some dicotyledonous plant species. Additionally, through the analysis of publicly available insect transcriptomic databases, numerous insect virus-like sequences were discovered in insects. The coat proteins encoded by these novel viruses typically contain a conserved domain similar to that found in the coat protein of plant viruses in the *Virgaviridae* family. Interestingly, endogenous viral elements (EVEs) related to virga-like viruses were also found in insect genomes [14].

In this investigation, we identified two previously unknown RNA viruses, designated as the Cavariella aegopodii virga-like virus 1 (CAVLV1) and Cavariella aegopodii iflavirus 1 (CAIV1), in *C. aegopodii*. The complete genome sequence of CAVLV1 and nearly the entire genome sequence of CAIV1, including the 3′ untranslated region (UTR), have been successfully determined. Our analysis of small RNA (sRNA) data indicates the activation of the RNA interference (RNAi) antiviral immune pathway following the infection of RNA viruses.

## 2. Materials and Methods

### 2.1. Insect Sampling and Species Definition

The specimens of the willow-carrot aphid utilized in this study were collected from the same location in Yunnan province, China, in 2020. Subsequently, a group of about 15–20 aphids mixed together was homogenated with TRIzol (Invitrogen, Carlsbad, CA, USA) in liquid nitrogen and stored in a −80 °C refrigerator placed in our lab. To confirm the species identification of *C. aegopodii*, the mitochondrial cytochrome oxidase I (COI) gene was amplified, cloned, and validated via Sanger sequencing using a set of universal primers.

### 2.2. RNA Extraction and Sequencing

The insect RNA containing rRNA were generated referring to the reagent specification. The RNA concentration and integrity were then measured using Nanodrop (Thermo Scientific, Waltham, MA, USA), following which, high-throughput sequencing (HTS) and small RNA sequencing were performed. 

### 2.3. The cDNA and Small RNA Library Were Produced through the Illumina TruSeq

The total RNA with rRNA Sample Preparation Kit and the Truseq Small RNA Library Preparation Kit were used, respectively (Illumina, San Diego, CA, USA). Illumina HiSeq 2500 platform and Nova seq 6000 were selected to realize transcriptome and small RNA sequencing, respectively. Finally, 19 Mbp of raw data were completed and detected. The sequencing reads of the transcriptome datasets underwent a quality assessment using FastQC and Trimmomatic (version 0.39). Subsequently, the filtered reads were de novo assembled with Trinity (version 2.8.5) [15] using default parameters (-no_normalize_reads). The resultant assembled contigs were then compared to the NCBI viral RefSeq database using diamond (v2.0.15.153) Blastx. In addition, a total of 21.3 million paired-end reads were sequenced, and 2 × 150 bases were sequenced from the ends.

### 2.4. Virus Genome Verification and Small Interfering RNA Detection

The methodology for identifying potential virus genomes have been detailed in our previous literature [16]. The assembled RNA-seq clusters were aligned with the viral RefSeq database from NCBI via the BlastX method, and an E-value cutoff was set at 2 × 10^−20^ for each comparison. To minimize false positive matches, contigs similar to viruses and exceeding 3000 bp were searched sequentially against the NCBI’s complete nucleotide (NT) and non-redundant (NR) protein databases via Blastn and Blastx methods. The validation of identified virus-derived contigs was identified through RT-PCR, utilizing the primers specified in Supplementary Table S1.

In addition, to demonstrate whether the small interfering RNA (siRNA) pathway participated in antiviral defense in *C. aegopodii*, small RNA (sRNA) data derived from new viruses were analyzed through Linux bash scripts and custom Perl scripts. The script was downloaded from the website (https://github.com/Gyoungwe/novel_virus_analysis, accessed on 5 April 2024). And we changed the visual parameters and color scheme in the script. Small RNAs of 18–30 nt in length, corresponding to the whole genome of the virus, were sorted through applying Bowtie 1 software [17]; we used the strictest alignment and changed the value of -v to 0 in the Bowtie 1 script. 

### 2.5. Determination of Viral Genome Termini and Transcript Abundance

The complete genome sequence of the virus was determined through the rapid amplification of cDNA ends (RACE). The cDNAs of the genome end were generated using the SMARTer® RACE 5′/3′ kit and were subsequently amplified via PCR with gene-specific primers (GSPs) and a universal primer mixture (UPM). The PCR reaction (50 μL) included a 1.5 μL cDNA Adaptor, 1.5 μL 10 × UTM primer mix, 1.5 μL GSPs, 25 μL 2 × Phanata Buffer, 1 μL dNTP, and 1 μL Phanata Max Super-Fidelity DNA polymerase (Vazyme, Nanjing, China), with the addition of 20 μL ddH_2_O.

The PCR thermal cycling protocol was as follows: initial denaturation at 95 °C for 3 min, followed by 35 cycles of denaturation at 95 °C for 30 s, annealing at 65 °C for 30 s, and extension at 72 °C for 30 s. Subsequently, the PCR products were cloned into 5 × TA/Blunt-Zero Cloning (Vazyme, Nanjing, China), and then Sanger sequencing was carried out. The primers related to RACE and the verification of the virus genome are described in detail in Supplementary Table S1.

To assess the abundance and coverage of the virus, reads from the transcriptome were trimmed for adapters and for quality through Bowtie2 (v2.3.5.1) [16] and Samtools (v1.7)software [18], and were aligned against the complete viral genome. The matched reads coverage was visualized using an Integrated Genomics Viewer [19].

### 2.6. Virus Structure Analysis 

The ORF Finder tool (https://www.ncbi.nlm.nih.gov/orffinder/, accessed on 31 January 2024) and the NCBI Conserved Domain database (https://www.ncbi.nlm.nih.gov/cdd/, accessed on 31 January 2024) were selected to predict the open reading frames (ORFs) and the conserved protein domains, respectively.

### 2.7. Identification of the Virus Taxonomic Status 

The phylogenetic analyses were performed following anteriorly established protocols, with some refinements being made [20]. The amino acid sequence of the RNA-dependent RNA polymerase (RdRP) from all viruses were loaded and aligned to generate the evolutionary tree via the MAFFT (v7.487) method [21]. In addition, Gblock (0.91b) [22] was employed to trim ambiguous regions in the alignment. Subsequently, blocks that were divergent and ambiguously aligned were excluded from the protein sequence alignments to enhance the phylogenetic accuracy. ModelTest-NG was employed to ascertain the optimal model for amino acid substitutions [23]. Moreover, RAxML-NG (v. 0.9.0) [24] was used to construct the maximum likelihood (ML) tree, which was visualized and refined using iTOL [25]. The detailed virus RdRP sequences can be found in Supplementary Table S2.

## 3. Results

### 3.1. Discovery of New RNA Viruses Fragments in C. aegopodii

The total RNA of insects were handled with (HTS) to detect RNA virus-related sequences in *C. aegopodii*. One sample including 10–15 aphids was analyzed via RNA-Seq. Raw data were generated through deep sequencing on the Illumina HiSeq 4000 platform using Novogene. The aphid species was identified as *C. aegopodii*, showing 100% homology as compared to other COI sequences in the GenBank (accession number: OP956128.1). A total of 21,303,174 reads were produced, comprising 55,388 and 4261 reads corresponding to CAVLV1-like and CAIV1-like sequences, respectively. In addition, a total of 1,077,307 vsiRNA reads were analyzed, including 997 unique reads, and 2459 unique reads were perfectly mapped to the assembled CAIV1 and CAVLV1 genome sequence, respectively. 

The SRA data accession number is PRJNA1109727. Following BLASTn searches against the NCBI nucleotide (nt) and viral reference databases, two previously unidentified RNA viruses were discovered within the assembled contigs of *C. aegopodii* from the transcriptome data, being designated as CAVLV1 and CAIV1 (Table 1). The sequences of these two viruses were submitted to NCBI and assigned the accession numbers PP500773 and PP500774. The sequences of two new viruses were confirmed via RT-PCR with related primers and was demonstrated via Sanger sequencing (Supplementary Table S1).

### 3.2. The Genome Structure and RNA-Seq Read Coverage of CAVLV1 and CAIV1

**Figure 1:**
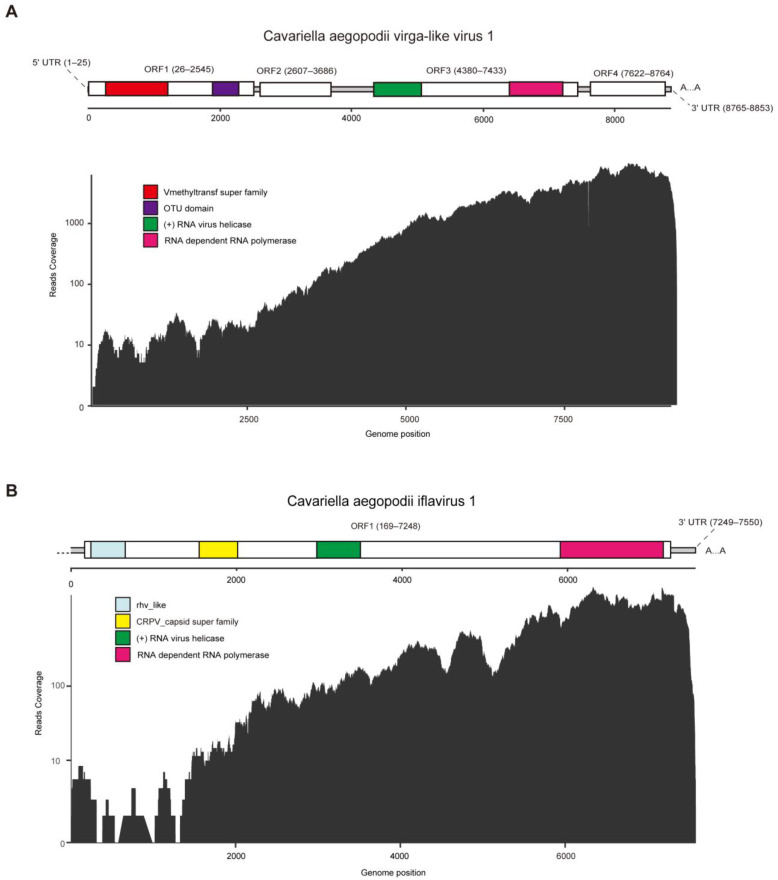
The genome structure and RNA-seq read coverage of CAVLV1 (**A**) and CAIV1 (**B**). The white frame shows ORFs. Different colored blocks represent different domains of protein; red represents the Vmethyltransf superfamily; purple represents the OTU domain; green represents the (+) RNA virus helicase; pink represents RNA-dependent RNA polymerase; blue represents rhv_like; and yellow represents CRPV_capsid super family. RHV, picornavirus capsid protein domains; CRPV, cricket paralysis virus; OTU, ovarian tumor.

The full-length genome sequence of CAVLV1 is 8853 nt, comprising a 5′ untranslated region (UTR) of 25 nt, a 3′-untranslated region (UTR) of 89 nt, and four predicted open reading frames (ORFs), ORF1 (26–2545 nt), ORF2 (2607–3686 nt), ORF3 (4380–7433 nt), and ORF4 (7622–8764 nt). Through InterProScan analysis, CAVLV1 was found to contain four conserved domains as follows: Vmethyltransf super family domain (266–1204 nt), OTU domain (1892–2293 nt), RNA virus helicase domain (4341–5057 nt), and an RNA-dependent RNA polymerase domain (RdRp, 6417–7223 nt) (Figure 1A). 

The verified genome sequence of CAIV1 include a 3′-untranslated region (UTR) of 302 nt and one predicted open reading frame (ORF) from 169 to 7248 nt, totaling 7550nt. According to the analysis of InterProScan, the ORF of CAIV1 have typical domains of iflaviruses, including the rhv_like domain (rhv, 241–660 nt), CRPV_capsid super family domain (CRPV, 1546–2022 nt), RNA virus helicase domain (3046–3369 nt), and an RNA-dependent RNA polymerase domain (RdRp, 6208–7149 nt) (Figure 1B). 

### 3.3. Phylogenetic Analysis of CAVLV1 and CAIV1

**Figure 2:**
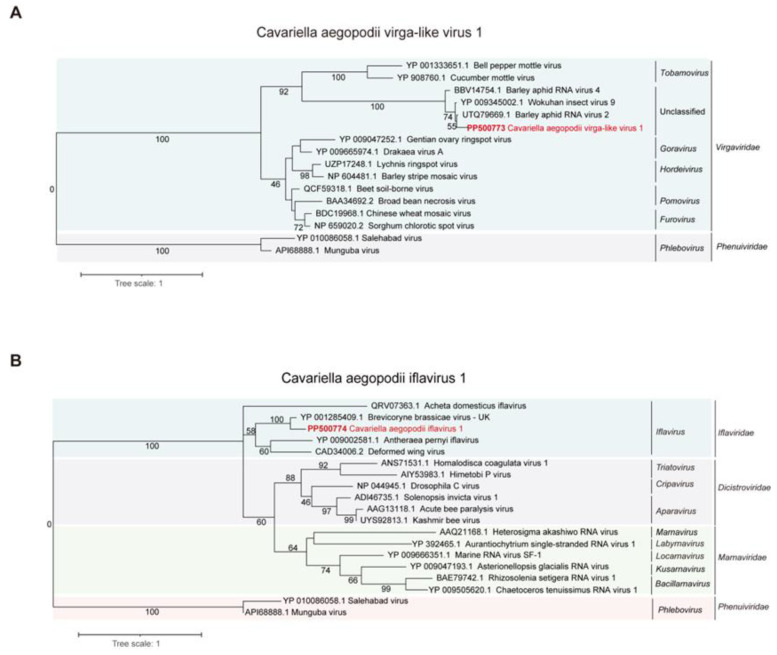
Phylogenetic analysis of CAVLV1 and CAIV1. (**A**) Evolutionary distance of CAVLV1 and other similar viruses classified in *Virgaviridae*. (**B**) Evolutionary distance of CAIV1 and other viruses attached to the *Marnaviridae*, *Dicistroviridae*, *Iflaviridae*, and *Phenuiviridae*. The two viruses of *Phenuiviridae* were used for the outgroup of the tree. Bootstrap data are depicted above the nodes of the trees. Scale bars indicate percentage divergence, and CAVLV1 and CAIV1 are highlighted in red font.

Phylogenetic analysis revealed a close relationship between CAVLV1 and the *Virgaviridae* family, particularly with Wuhan insect virus 9, Barley aphid RNA virus 4, and Barley aphid RNA virus 2 (Figure 2A). Phylogenetic analysis indicated that CAIV1 clustered closely with the Brevicoryne brassicae virus UK, Antheraea pernyi iflavirus, and Deformed wing virus, suggesting a close relationship with the *Iflaviridae* family (Figure 2B).

### 3.4. SiRNA Analyses of CAVLV1 and CAIV1

**Figure 3:**
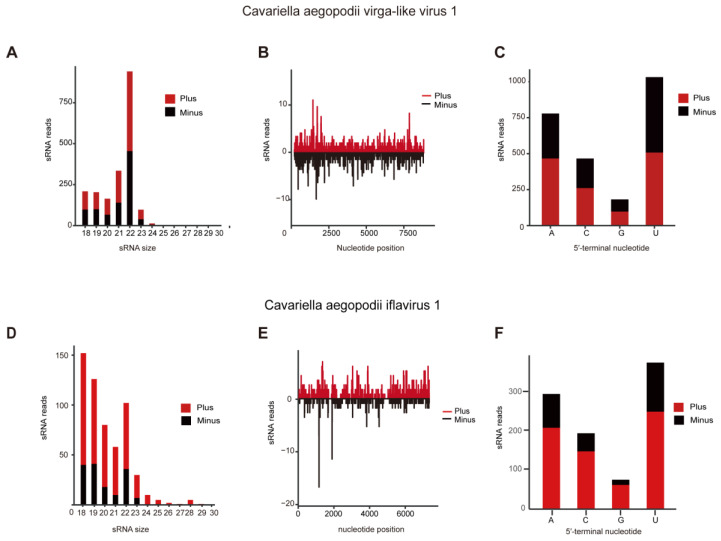
The siRNA analysis of CAVLV1 and CAIV1. (**A**) The characteristic and amount of sRNA derived from CAVLV1. (**B**) Presence of sRNA produced by CAVLV1 on the whole genome. (**C**) Preference of 5′-end sequence of CAVLV1. (**D**) The characteristic and amount of sRNA derived from CAIV1. (**E**) Presence of sRNA generated by CAIV1 on the entire genome. (**F**) Preference of 5′-end sequence of CAIV1.

CAVLV1 showed efficient replication in C. aegopodii, as evidenced by its extensive read coverage across the entire genome and the presence of small RNAs (Figure 3B). In terms of vsiRNA, CAVLV1 induced vsiRNA production with peaks at 22 nt (Figure 3A), leading to the generation of untranslated areas and asymmetrical hotspots on positive and negative senses across the entire genome of CAVLV1 (Figure 3C). The viral siRNAs of CAVLV1 exhibited an obvious preference for the A/U in 5′-tail end of the genome (Figure 3C). Similarly, CAIV1 exhibited efficient replication in C. aegopodii, as evidenced by its read coverage spanning the entire genome and the presence of small RNAs (Figure 3E,F). The characteristics of siRNA in C. aegopodii suggested that the RNAi pathway plays a significant role in resisting ISVs infection. However, CAIV1 induced peaks at 18 nt in vsiRNA reads (Figure 3D), with untranslated areas and asymmetric hotspots on positive and negative sense. Further research is needed to confirm the role of the siRNA characteristics of CAIV1. 

### 3.5. The Evolutionary Distances of between CAVLV1 or CAIV1 and Other Similar Viruses

**Figure 4:**
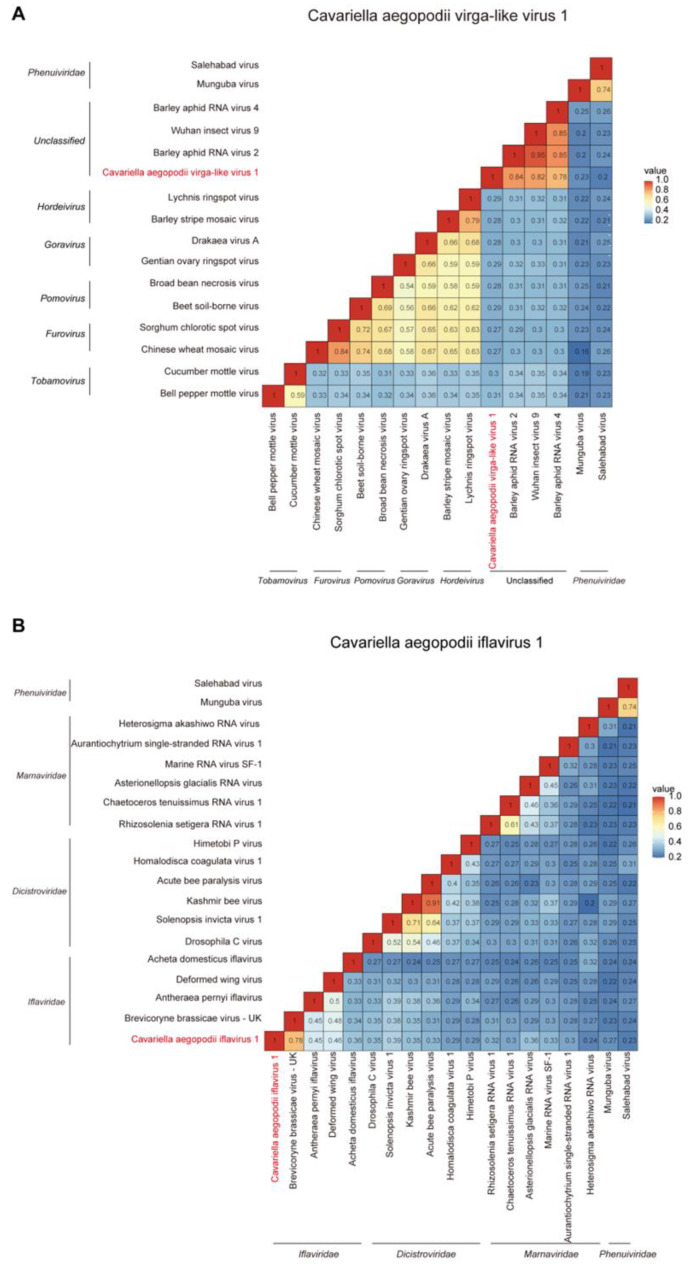
The evolutionary distances analysis of CAVLV1 (**A**) and CAIV1 (**B**) referred to sequences from RdRp. The red font color represents two new viruses in this study.

The evolutionary distance analysis results concerning RdRp showed that CAVLV1 has the closest homologous relation with the Wuhan insect virus 9, Barley aphid RNA virus 4, and Barley aphid RNA virus 2. The identity was 80–90% (Figure 4A). Additionally, the specific evolutionary distances between CAIV1 and the selected viruses in the phylogenetic tree were also visualized. CAIV1 has the closest homologous relation with the Brevicoryne brassicae virus UK, Antheraea pernyi iflavirus, and Deformed wing virus. The identity was 30–75% (Figure 4B).

## 4. Discussion

With the advancement of high-throughput sequencing and bioinformatics analysis, numerous RNA viruses have been identified, providing deeper insights into insect viromes and viral evolution. Recently, a large number of RNA viruses have been found in a variety of aphids, such as *Myzus persicae* [27]. In our work, two new insect RNA viruses, called asCAVLV1 and CAIV1were identified in *C. aegopodii*. Rod-shaped virions are characteristic of most virga viruses, with the exception of tobamo viruses (genus *Tobamovirus*), for which the vectors remain unknown. These viruses are primarily transmitted by soil-dwelling organisms, such as plasmodiophorid protists or nematodes. The hosts are mainly plants, but some virga-like viruses have also been reported in insects [14]. 

Meanwhile, Virga viruses generally have three characteristics, including the length of the genome being classified as ssRNA (+), the length being about 6300–13,000 nt, and the genome encoding three ORFs [10,28]. Iflaviruses are a type of insect virus that lack an outer envelope. They carry a single-stranded RNA genome with a positive polarity (+), featuring polyadenylated tails at the 3′ untranslated region (UTR). These viruses encode a large polyprotein, with the viral coat protein being situated in the N-terminal domain. The C-terminal region houses non-structural proteins which are responsible for replication and polyprotein processing [29].

In our study, the genomic features of CAVLV1 are consistent with most Virga viruses, possessing four ORFs (Figure 1A). CAIV1 is similar to other Iflaviruses, with only one ORF for a large polyprotein that encodes proteins with CRPV and RdRP functional domains (Figure 1B). 

As potential biocontrol agents, ISVs have attracted increasing attention for their ability to affect vectoring abilities. They have been found to inhibit the infection of certain arboviruses, such as **Nhumirim virus** and **Zika virus**, both in vivo and in vitro in mosquitoes [30]. ISVs are also involved in decreasing the spread of **West Nile virus** in coinfecting mosquitoes. However, their effectiveness can be hindered by **Menghaic rhabdovirus** and **Shinobi tetravirus** when infecting the *Aedes albopictus* C6/36 cell line [30,31]. Furthermore, ISVs are known to modulate the innate immune pathway in certain insects, including mosquitoes, which in turn hinders viral replication and reduces vector competence. ISVs mainly regulate the insect immune pathway by activating the RNA interference (RNAi) pathway of the host cell, thus producing small RNAs when infecting insects. These small RNAs can bind to viral RNA complementarily, leading to the degradation of the viral genome, thereby inhibiting viral replication and transmission [32]. In our study, we observed that CAVLV1 may participate in antiviral response in *C.aegopodii* during virus infection. It is groundless for the antiviral function of CAIV1. 

Summarily, two new viruses, named CAVLV1 and CAIV1, were discovered in *C. aegopodii.* And they were listed in the category of virga-like viruses and iflavirus based on phylogenetic analysis, respectively. These two viruses all produced siRNA, which may activate RNAi to resist viruses in *C. aegopodii*. Our research will help to increase the abundance of viruses in aphids, offering valuable information regarding the coevolution between virga-like viruses or iflavirus and aphids.

## Figures and Tables

**Table 1:** The detailed messages concerning the two novel viruses identified in *Cavariella aegopodii*.

Virus Names	Accession	Length (nt)	Classification	Abundance	E-Value	RdRP Protein Identities	Top BlastP Hit Virus	Virus Reference
Cavariella aegopodii virga-like virus 1 (CAVLV1)	PP500773	8853	virga-like virus	2459	0.0	79.29%	Hubei Wuhan insect virus 9	[2]
Cavariella aegopodii iflavirus 1 (CAIV1)	PP500774	7550	Iflavirus	997	0.0	74.26%	Brevicoryne brassicae virus–UK	[26]

## Data Availability

The data presented in this study are available within the article and Supplementary Materials.

## Supplementary Materials

The following supporting information can be downloaded at: https://www.mdpi.com/article/10.3390/v16060919/s1. Table S1: Primers used for RT-PCR in this study; Table S2: Abbreviations of virus names and GenBank accession numbers used in this study.
