# Supplementary material for: Discovery of Two Novel Viruses of the Willow-Carrot Aphid, Cavariella aegopodii
Source: Viruses. 2024 Jun 5;16(6):919. doi: 10.3390/v16060919 (PMC11209057; doi:10.3390/v16060919)
Supplement: Supplementary file 1 [file viruses-16-00919-s001.zip › viruses-2983886-supplementary/Supplementary Files/Supplementary Table S1.pdf]

Table S1. Primers used for RT-PCR in this study

Primers used for RT-PCR	Forward primer (5’-3’)	Reverse primer (5’-3’)
**Cavariella aegopodii virga-like virvs 1 (CAVLV1)**		
CAVLV1 5′-RACE GSP	AATAAGTCTTGATCTTCCTGACTCA	CTAATACGACTCACTATAGGGCAAGCAGTGGTATCAACGCAGAGT
CAVLV1 3′-RACE GSP	GTCCCGATGTCAAATATCATGAT	CTAATACGACTCACTATAGGGC
CAVLV1 segment1	CTGACGCGCTGATGACCCAAGTT	CATCGAGACGTATGGCATAATGTC
CAVLV1 segment2	ATTTATTAACGACTCGCAACCGGCC	CATCATAATCAAAAAGTTGCACATC
CAVLV1 segment3	CAATCACTGTATAGGGTACCATCCG	CAATCTTCAATATCGTCGGATTCTG
CAVLV1 Segment4	TGACGTACAAGGTCCAATAGATGTC	CACACGCCATGACGTATGTAGAATC
CAVLV1 Segment5	CGTCTTTCTTTCCAAATTTAGATTC	AGAGCGTTAACTTGATTATCAGAAG
CAVLV1Segment6	CTGATAATCAAGTTAACGCTCTA	GCCACTACTATAGTTTCATCGTTGT
CAVLV1 Segment7	CTTCTGATAATCAAGTTAACGCTCT	GACTTCCCTGAAACATACATTCT
CAVLV1 Segment8	GTATCCTATAGATGTCCGCAAGATG	TATGGTTGTCGGTACTTTCAATTAC
CAVLV1 Segment9	ACAGATAAACGCAGTTGATTATGCC	GTCTTTAGGAGTAAAGACAACGGTT
CAVLV1 Segment10	GTTGTTTACGACGATACCAATCATG	GACAGTGCACCGAAATCCGTATCAG
CAVLV1 Segment11	CACGTCACTTTACTTTTGTTCAAGG	CCTTGAACAAAAGTAAAGTGACGTG
CAVLV1Segment12	GTTTACCACCATTGAATAGGTACAA	CTAATACGACTCACTATAGGGC
**Cavariella aegopodii iflavirus 1 (CAIV1)**		
CAIV1 3′-RACE GSP	GCTTGAGGAAGCTAGCATCACTGAG	CTAATACGACTCACTATAGGGC
CAIV1 segment1	GACCACTAGGAATTGATATAC	GTATTACACTTTAGAAGG
CAIV1 segment2	CGGAGAGAGATTTGATGATCTGAAG	CAGCCTTATCAACTAAAGTGTCTAC
CAIV1 segment3	CCCGTCCACTATTAAATATAAGGCT	GAGCGTAAGCCACTCTTGGATAG
CAIV1 egment4	TATAGCTAAGGGAACTAGTCAAGCT	CAGAATACATAGTATTGGGATCCCT

CAIV1 segment5	GTGTATCCGTTTGGTTTTAATGATG	GCAGTAAGGTAATAACATGTGAACA
CAIV1 segment6	TTGTACATTCCCTACAAATGTTACC	TGGCTCCAGATATTGGTGTAACTAC
CAIV1 segment7	GATTACACTATAACTTGGTGTAAGA	GACAATCGTCATGTATTGTCAAAGG
CAIV1 segment8	CGTCCCTTAATCTGGAATACCTCTG	GCTAGGTACAAACCCTTTCGTTTAG
