# Supplementary material for: Discovery of Two Novel Viruses of the Willow-Carrot Aphid, Cavariella aegopodii
Source: Viruses. 2024 Jun 5;16(6):919. doi: 10.3390/v16060919 (PMC11209057; doi:10.3390/v16060919)
Supplement: Supplementary file 1 [file viruses-16-00919-s001.zip › viruses-2983886-supplementary/Supplementary Files/Supplementary Table S2.pdf]

Table S2. Abbreviations of virus names and GenBank accession numbers used in this study

Abbreviations	Complete virus name	Accession (genome)	Accession (RdRp)
Virgaviridae			
Furovirus			
CWMV	Chinese wheat mosaic virus	LC657081.1	BDC19968.1
SCSV	Sorghum chlorotic spot virus	NC_004015.1	NP 659020.2
Pomovirus			
BSBV	Beet soil-borne virus	MK731956.1	QCF59318.1
BBNV	Broad bean necrosis virus	NC_004425.1	BAA34692.2
Goravirus			
GORV	Gentian ovary ringspot virus	NC_024502.1	YP 009047252.1
DVA	Drakaea virus A		YP 009665974.1
Hordeivirus			
BSMV	Barley stripe mosaic virus	U13918.1	NP 604481.1
LRSV	Lychnis ringspot virus	MG913811.1	UZP17248.1
Tobamovirus			
BPeMV	Bell pepper mottle virus	MG913811.1	YP 001333651.1
CuMoV	Cucumber mottle virus	NC_008614.1	YP 908760.1
Unclassified			
CAVLV1	Cavariella aegopodii virga-like virus 1	PP500773	
BARV4	Barley aphid RNA virus 4	LC516838.1	BBV14754.1
WIV9	Wuhan insect virus 9	NC_033710.1	YP 009345002.1
BARV2	Barley aphid RNA virus 2	LC516836.1	UTQ79669.1
Marnaviridae			

Bacillarnavirus			
RsRNAV01	Rhizosolenia setigera RNA virus 01	NC_018613.1	BAE79742.1
CtenRNAV01	Chaetoceros tenuissimus RNA virus 01	NC_038321.1	YP 009505620.1
Kusarnavirus			
AglaRNAV	Asterionellopsis glacialis RNA virus	NC_024489.1	YP 009047193.1
Locarnavirus			
MRVSF-1	Marine RNA virus SF-1	NC_043515.1	YP 009666351.1
Labyrnavirus			
AssRNAV01	Aurantiochytrium single-stranded RNA virus 01	AB193726.1	YP 392465.1
Marnavirus			
HaRNAV	Heterosigma akashiwo RNA virus	AY285768.1	AAQ21168.1
Dicistroviridae			
Aparavirus			
SINV1	Solenopsis invicta virus 1	DQ855655.1	ADI46735.1
KBV	Kashmir bee virus	KC130158.1	UYS92813.1
ABPV	Acute bee paralysis virus	OK170033.1	AAG13118.1
Cripavirus			
DCV	Drosophila C virus	NC_001834.1	NP 044945.1
Triatovirus			
HoCV1	Homalodisca coagulata virus 1	KT207917.1	ANS71531.1
HiPV	Himetobi P virus	MT603635.1	AIY53983.1
Iflaviridae			
Iflavirus			
CAIV1	Cavariella aegopodii iflavirus 1	PP500774	
AdIV	Acheta domesticus iflavirus	NC_078648.1	QRV07363.1
BrBV	Brevicoryne brassicae virus - UK	NC_009530.1	YP 001285409.1

ApIV	Antheraea pernyi iflavirus	NC_023483.1	YP 009002581.1
DWV	Deformed wing virus	JF346657.1	CAD34006.2
Phenuiviridae			
SALV	Salehabad virus	NC_055296.1	YP_010086058.1
MUNV	Munguba virus	KX611396.1	API68888.1
